# Respiratory distress syndrome prediction at birth by optical skin maturity assessment and machine learning models for limited-resource settings: a development and validation study

**DOI:** 10.3389/fped.2023.1264527

**Published:** 2023-11-15

**Authors:** Zilma Silveira Nogueira Reis, Gisele Lobo Pappa, Paulo de Jesus H. Nader, Marynea Silva do Vale, Gabriela Silveira Neves, Gabriela Luiza Nogueira Vitral, Nilza Mussagy, Ivana Mara Norberto Dias, Roberta Maia de Castro Romanelli

**Affiliations:** ^1^ Faculdade de Medicina, Universidade Federal de Minas Gerais, Belo Horizonte, Brazil; ^2^ Departamento de Ciência da Computação, Universidade Federal de Minas Gerais, Belo Horizonte, Brazil; ^3^ Pediatrics and Neonatology Department, University Hospital, ULBRA, Canoas, Brazil; ^4^ Neonatal Intensive Care Unit, University Hospital, UFMA, São Luis, Brazil; ^5^ Hospital Sofia Feldman, Belo Horizonte, Brazil; ^6^ Faculdade de Medicina da Ciências Médicas de Minas Gerais, Belo Horizonte, Brazil; ^7^ Hospital Central de Maputo, Maputo, Mozambique

**Keywords:** respiratory distress syndrome, newborn, prematurity, childbirth, skin physiological phenomena, machine learning, equipment and supplies, medical device

## Abstract

**Background:**

A handheld optical device was developed to evaluate a newborn's skin maturity by assessing the photobiological properties of the tissue and processing it with other variables to predict early neonatal prognosis related to prematurity. This study assessed the device's ability to predict respiratory distress syndrome (RDS).

**Methods:**

To assess the device's utility we enrolled newborns at childbirth in six urban perinatal centers from two multicenter single-blinded clinical trials. All newborns had inpatient follow-up until 72 h of life. We trained supervised machine learning models with data from 780 newborns in a Brazilian trial and provided external validation with data from 305 low-birth-weight newborns from another trial that assessed Brazilian and Mozambican newborns. The index test measured skin optical reflection with an optical sensor and adjusted acquired values with clinical variables such as birth weight and prenatal corticoid exposition for lung maturity, maternal diabetes, and hypertensive disturbances. The performance of the models was evaluated using intrasample k-parts cross-validation and external validation in an independent sample.

**Results:**

Models adjusting three predictors (skin reflection, birth weight, and antenatal corticoid exposure) or five predictors had a similar performance, including or not maternal diabetes and hypertensive diseases. The best global accuracy was 89.7 (95% CI: 87.4 to 91.8, with a high sensitivity of 85.6% (80.2 to 90.0) and specificity of 91.3% (95% CI: 88.7 to 93.5). The test correctly discriminated RDS newborns in external validation, with 82.3% (95% CI: 77.5 to 86.4) accuracy. Our findings demonstrate a new way to assess a newborn's lung maturity, providing potential opportunities for earlier and more effective care.

**Trial registration:**

RBR-3f5bm5 (online access: http://www.ensaiosclinicos.gov.br/rg/RBR-3f5bm5/), and RBR-33mjf (online access: https://ensaiosclinicos.gov.br/rg/RBR-33rnjf/).

## Introduction

Infant mortality is a critical human development indicator since it reflects the quality of assistance, and social, economic, and environmental factors (1). Most child deaths occur due to prematurity meeting lung immaturity as the main bare reason (2). Approximately 11% of newborns worldwide are preterm, born earlier than 37 weeks of gestational age, and of whom 6% are late preterm, born between 34 and 37 weeks of gestational age (3) and require specialized care (4). Respiratory distress syndrome (RDS) is a common reason for neonatal intensive care unit (NICU) admission and neonatal mortality. Since lung immaturity due to surfactant deficiency is the cause of the disease, respiratory failure occurs soon after birth. However, most respiratory insufficiency at birth is not accurately evaluated, leading to poor outcomes because of delays in appropriate treatment (4, 5). Indeed, on many occasions, the respiratory picture at birth can be confused with an adaptive syndrome such as transient tachypnea of the newborn (TTN), as well as non-respiratory reasons, which may be cardiac, neurological, metabolic, or hematological, among others (6). Clinical history, lung image assessment, and blood lab tests are clues to discriminate between RDS and other respiratory distress, pointing newborns at higher risks of severe complications (7). Beyond clinical manifestation, assessing lung maturity is supported by biochemical and biophysical tests on amniotic fluid, genetic approaches, and microbubble evaluation in gastric aspirates (8). Unfortunately, the lack of healthcare technologies increases exponentially in low- and middle-income countries (LMICs) in scenarios with limited neonatal assistance, where the burden of preterm birth is higher than in other countries (4).

To achieve lower infant morbidity and mortality rates focused on the day of birth, early identification of lung maturity risk enhances chances of survival even based on referral safe transportation among facilities. Nevertheless, very often, especially late preterm infants are inappropriately classified as full-term newborns, delaying care for the former (9). This way, improvements centered on equity of technology access and quality of antenatal and childbirth care can reduce neonatal health disparities among birth scenarios with or without full support for preterm children identification and treatment (1, 10). The search for an affordable approach to quickly identify premature infants according to the degree of lung maturity remains a relevant target for health systems. Early intervention to manage respiratory distress in a newborn could mean the difference between survival and, possibly, a reduction in mortality (11).

Lungs develop linearly before childbirth; however, the maturational competence for extrauterine breathing occurs later in pregnancy or under stressful influences such as maternal disease, placental dysfunction, and drug exposition (12). Under the scientific basis, evidence is extensive concerning the influence of corticosteroid exposition during the prenatal period to prepare fetuses for after-birth life (13). At the same time, the skin is a tissue with late maturation, postponing the protective external barrier to near-term and term gestation (14, 15). Meanwhile, there is a direct relationship between epidermal layer competence and neonatal survival, facing risks of hypothermia, water loss, and infections (16, 17). Likewise, in this organ, antenatal corticotherapy induces cytodifferentiation and keratinization, enhancing the chances of survival (13). Beyond visual inspection of skin appearance, which characterizes preterm newborns (18), an objective measure of skin reflectance with a photometer was correlated with gestational age (19). Based on a multicenter clinical trial, a new medical device was able to assess the gestational age by adjusting a machine learning model for optical skin maturity to antenatal corticosteroid therapy for fetal maturation (ACTFM) and birth weight, discriminating preterm from term newborns, with 37 weeks of gestational age or more, with an area under ROC curve of 0.970, [95% CI: 0.959–0.981] (20). The present study explored new machine learning algorithms on the same optical device, to evaluate its ability to predict RDS in the first 72 h of life, even in places with scarce resources.

## Methods

### Cohorts

We analyzed two birth scenarios, one to provide predictive models and the other to apply them to a more realistic picture of the usage of the model. Accordingly, both studies were multicenter prospective, concurrent cohorts comprised of six urban referral perinatal centers. Five Brazilian urban referral centers for high-complexity perinatal care took part in the study: Clinical Hospital—Universidade Federal de Minas Gerais (as coordinator), Minas Gerais State; Sofia Feldman Hospital—Minas Gerais State; Hospital da Universidade Luterana do Brasil—Rio Grande do Sul State; Hospital Materno-infantil de Brasília—Federal District; and Hospital Universitário da Universidade Federal do Maranhão—Maranhão State. One referral center in Mozambique, the Maputo Central Hospital, the largest in the country, is headquartered in its capital.

**Figure 1:**
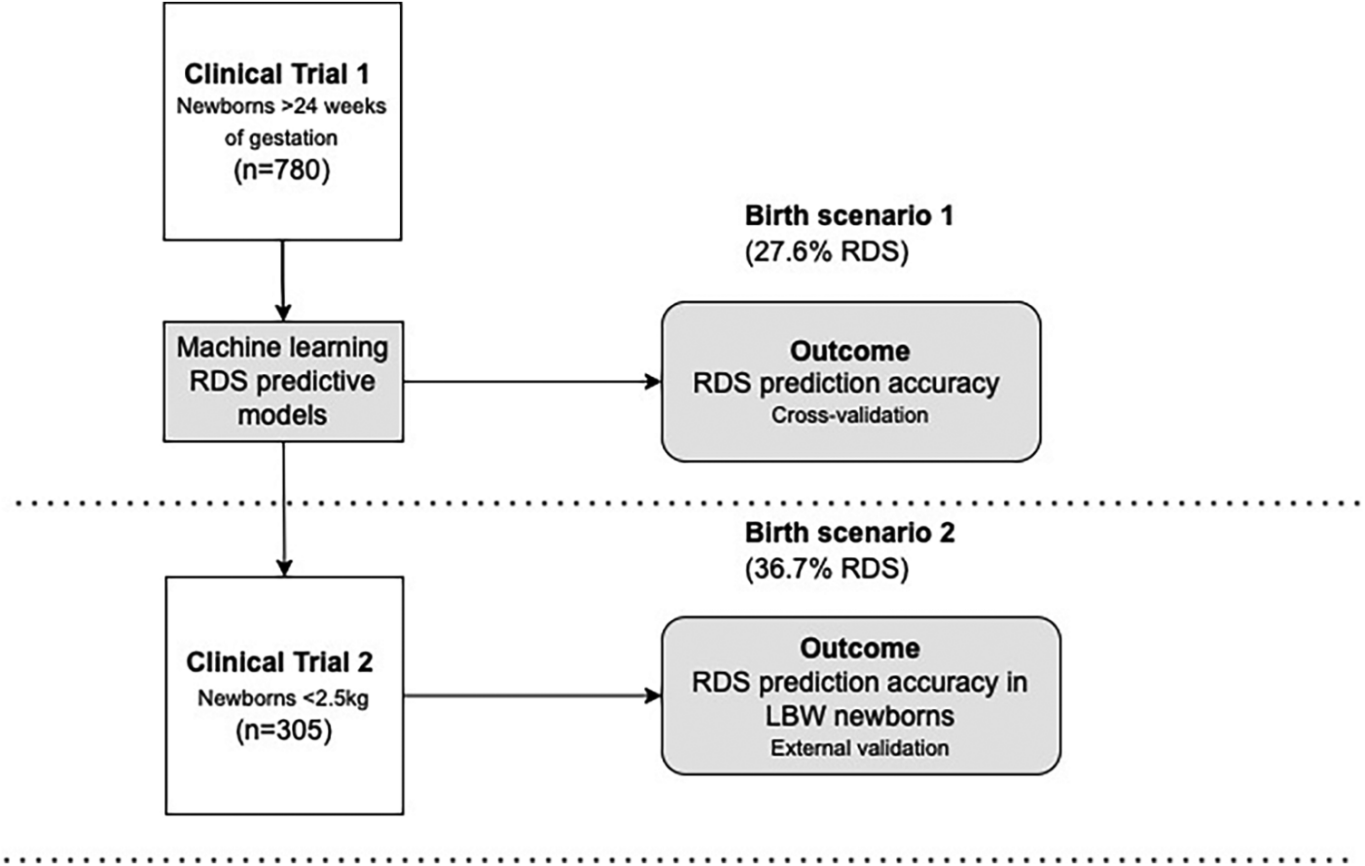
Database, birth scenarios, and index test (outcomes). LBW, low birth weight; RDS, respiratory distress syndrome.

Both cohorts shared inclusion criteria for live newborns enrolled within the first day of life, with the available reference standard gestational age, and childbirth after 24 weeks of gestation. Combining the last menstrual period with obstetric ultrasound assessment, we assessed gestational age at birth following international consensus for the due date (21). Anhydramnios, edema, congenital skin diseases, or chorioamnionitis were the exclusion criteria because they could modify skin structure, affecting the optical properties of the tissues. Teams of trained and certified health professionals and health professionals' research assistants enrolled and evaluated skin optical reflectance and clinical data at birth. All newborns had inpatient follow-up within the first 72 h of life to monitor immediate neonatal outcomes, with an early ending when discharge or death occurred, according to clinical trial protocols deposited in protocolos.IO (22). However, differences between the clinical characteristics of the newborns express different realities provided by birth weight eligibility criteria below 2.5 kg in the validation cohort (Figure 1).

For transparency, the clinical trials register and details of enrollment remain public. From clinical trial 1, registered under the number RBR-3f5bm5 (23), we evaluated Brazilian newborns with a gestational age of 24 weeks, and with any birth weight. The enrollment occurred from 2 January 2019 to 30 May 2021. Data from this study grounded the modeling process of machine learning prediction, thus being the baseline cohort. From clinical trial 2, registered under the number RBR-33rnjf (24), we assessed only newborns with birth weights under 2.5 kg in Brazil and Mozambique. The enrollment occurred from 15 February 2019 to 11 December 2021, and the dataset was used as the validation cohort. Most of the newborns were Mozambican (*n* = 177, 58.0%).

### Primary outcome

The primary outcome was to predict the RDS. The reference standard for RDS diagnosis has a basis in clinical, laboratory, and radiological findings and respiratory outcomes (7). However, concerning the reference standard in the scenario of LBW Mozambican newborns, when a radiological exam was absent, the diagnosis was based on clinical evaluations such as tachypnea, nasal flaring, retractions, and grunting with the possibility of progress to respiratory failure (24). In such a scenario where propaedeutics and other resources are unavailable, maternal and delivery context and clinical progress of respiratory failure were considered, based on clinical priority in 72 h of follow-up. Transient tachypnea of the newborn (TTN) was a differential diagnosis of respiratory complications at birth. Despite RDS being the target outcome, we introduced an exploratory modeling step by discriminating between RDS, TTN, or none. The diagnosis had a basis in clinical findings and respiratory outcomes (7). Again, TTN was diagnosed for exclusion in the Mozambican center, typically with clinical evidence of tachypnea shortly after birth, grunting, nasal flaring, retractions, and occasionally cyanosis (24). The procedures for clinical evaluation, complementary exams of the newborn, and RDS diagnosis are available in the Supplementary Material. Subgroups of analysis, according to LBW and very-LBW newborns, with a birth weight of less than 2.5 Kg and 1.5 Kg, respectively, provided a potential picture of the application according to ranges of birth weight.

### The index test

The assessment of newborns' skin maturity with the optical device was possible with the development of the equipment. We already noticed a high agreement between gestational age calculated by this device with the best available gestational age as a reference, as well the accuracy for discrimination of preterm against term infants (24). The error of the optical component had a prior evaluation, resulting in an intraobserver error of 1.97% (95% CI: 1.84–2.11) and an interobserver error of 2.6% (95% CI: 2.1–3.1) (24). The present analysis focused on RDS prediction as an additional value beyond the gestational age. Here, the index test was intended to analyze newborn lung maturity, clinically represented by RDS, as an unprecedented association with the optical skin maturity measurement in a machine learning algorithm.

**Figure 2:**
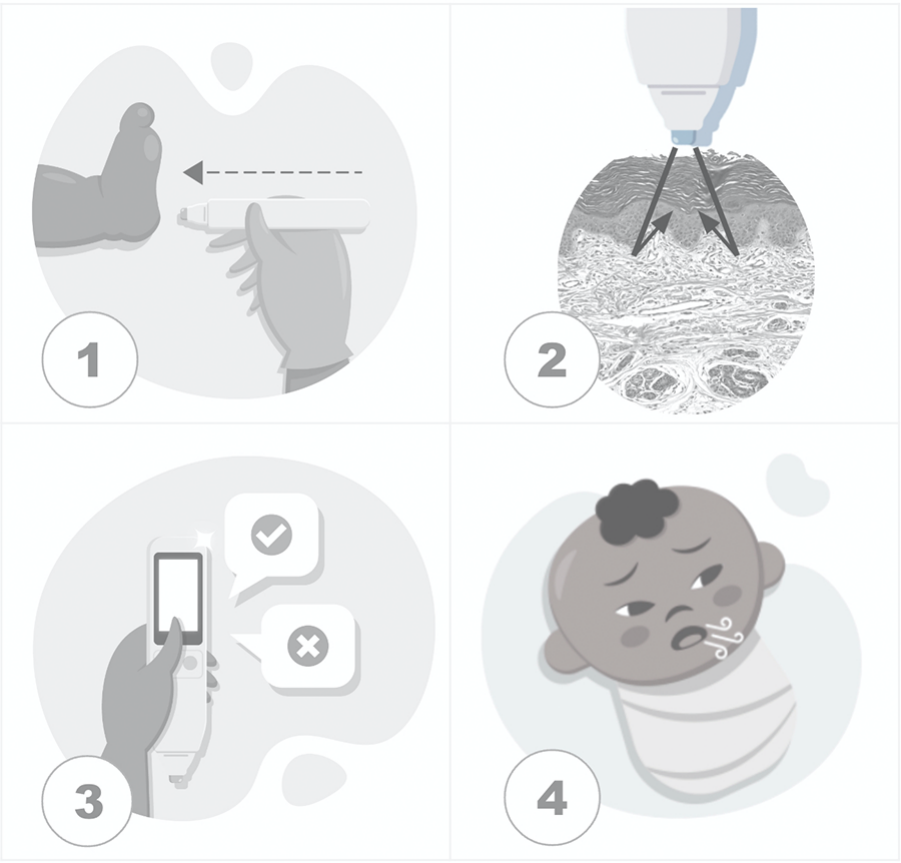
Steps of the testing process. (1) The device touches the skin over the sole of a newborn. (2) The sensor acquires skin maturity by assessing the photobiological properties of the tissue when measuring the reflection portions of the light beam incident on the skin. (3) The user inputs clinical data. (4) The data processor uses machine learning algorithms to predict respiratory distress syndrome.

In this study, data temporality of predictors was the first day of life, a moment when the user did not receive the result of RDS prediction to provide test blinding. Alongside skin reflectance, automatically acquired with the device when it touches the sole of the newborn, clinical variables were added by the user, and machine learning algorithms delivered the RDS prediction and were stored in the processor (Figure 2). In the future, the RDS prediction will be available on the device's screen.

The testing steps were standardized and supported by the prior proof of concept publications. The sole was the site of the newborn's body with a higher correlation between the skin reflection and pregnancy dating than other body sites, with the advantage of fulfilling the patient security recommendation for minimum manipulation of newborns (19). The influence of skin color and environmental conditions such as humidity, temperature, and ambient light were reasons for enhanced sensor design, achieving a prediction model without its adjustments (19, 25). This approach to newborns attended to requirements of patient security, including disinfection of the device with alcohol 70, and minimum manipulation of the child anywhere they were: inside incubators, warm crib, or in the mother's lap.

### Standard and data collection

According to recommendations for good clinical practices involving human research with medical devices, and according to the International Organization for Standardization (ISO 14155:2011), trained research assistants collected data on 65 demographic and clinical features and 25 skin variables. The framework of variables is available in a previous report (20). Clinical information was collected through structured questionnaires using software developed for the clinical trials, and, simultaneously, in paper formularies containing the exact requests. The data curation process double-checked the data from paper and electronic collection conducted by senior researchers, before opening the outcome blinding. Data consistency and completeness resulted in only one exclusion.

### Data availability

Data is available upon reasonable request and after anonymization to ensure ethical and legal data sharing, thus preserving the confidentiality of the persons who participated in this study.

### Ethics and dissemination

The studies involving humans had independent ethical board approval at each hospital. The Brazilian National Research Council approved the clinical trials under numbers 81347817.6.1001.5149 and 91134218.4.0000.5149. In Mozambique, ethical approval was under the number IRB00002657, according to the National Bioethics Council. Parents signed an informed consent form on behalf of the newborns as recommended by the Regulatory Bodies for Good Clinical Research Practice, and copies were retained in case they should be needed. Patients were not involved in the design of clinical trials. However, participants' parents received oral explanations and a press-illustrated folder with the proposal of the studies. Besides scientific articles, the results are continuously disseminated by non-scientific publications in media and on the project website: http://skinage.medicina.ufmg.br.

### Methods for estimating or comparing measures of diagnostic accuracy

#### Model development

We trained the models to binary prediction of RDS occurrence until 72 h of life with the five variables, and, additionally, for RDS, TTN, or none. The variables were: skin reflection, birth weight, ACTMF, diabetes, and hypertensive disturbances. The choice of independent variables took into account the easy access to data in the delivery scenario, the biological plausibility, and the importance-feature graphic analysis. Furthermore, we compared models based on three or five independent variables, including or not including maternal diseases. A wide range of models was tested, and the best results were obtained by the XGBoost Regressor model (26).

#### Model validation

The model was created using data from Clinical Trial 1. Two experiments were performed. In the first one, a ten-fold cross-validation procedure was used to assess the robustness of the model. This procedure was repeated 30 times, generating a total of 300 models that had their metrics of accuracy averaged and reported together with confidence intervals. The second experiment used data from Clinical Trial 1 to generate the model and from Clinical Trial 2 to validate the model.

#### Statistical analysis

For descriptive analysis of variables, we used average (SD) and median (IQR) to describe continuous variables for symmetric and asymmetric distributions, respectively. We used frequencies (%) for categorical variables. The Mean-T and Mann-Whitney U tests were used to compare the mean or median between two groups of interest as RDS yes or no, according to the variables’ parametric or non-parametric frequency distribution. For comparisons between frequencies, the Chi-square Test evaluated the independence hypothesis between categorical variables as preterm vs. RDS yes or no, and the Likelihood ratio chi-square statistic was the alternative when more than 20% of expected values were above five. ANOVA or Kruskall Wallis tests compared three groups analysis as RDS, TTN, and none according to the variables' parametric or non-parametric frequency distribution.

The set of machine learning models provided outcomes for binary RDS (yes or no) and three classes (RDS, TTN, none). The choice of the best models occurred by means of reliability analysis. The accuracy of the prediction of best models was evaluated using sensitivity, specificity, positive predictive value, negative predictive value, positive likelihood ratio, and negative likelihood ratio. *P*-values of <0.05 were considered suggestive of statistical significance. SPSS software (version 19.0; IBM Corp) was used for statistical data analysis.

## Results

### Description of newborns

**Figure 3:**
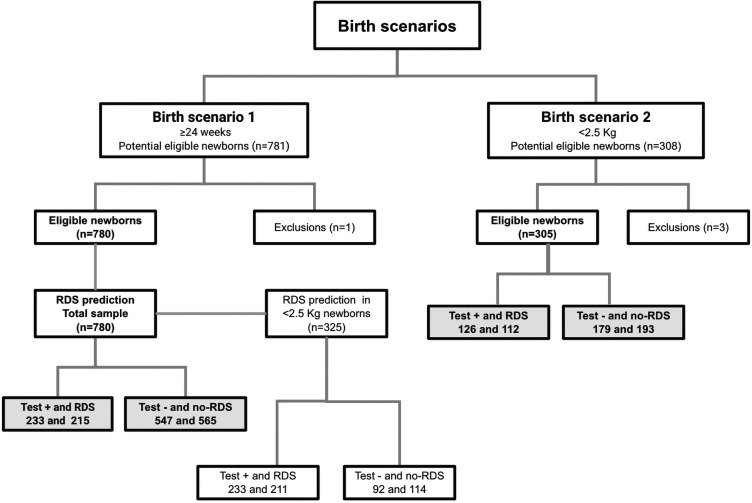
Flowchart of participants using STARD diagram, according to development and model validation birth scenarios.

Newborns from two clinical trials summed up 1,085 tests with the medical device. From the baseline scenario dataset where we set the RDS predictive models, we analyzed data from 702 Brazilian pregnant women who gave birth to 781 newborns with gestational ages older than 24 weeks (scenario 1). One exclusion occurred due to uncertainty in either an TTN or RDS diagnosis. Among 780 included newborns, 325 (41.7%) were low-birth-weight (LBW), and 27.6% (*n* = 215) had RDS. In the validation scenario, we analyzed data from 263 pregnant women who gave birth to 308 newborns with birth weights under 2.5 kg (scenario 2). Three exclusions occurred due to incorrect enrollment. Among the 305 included newborns, 37.7% (*n* = 112) had RDS. An overview of participants, according to development and model validation steps with respective birth scenarios and test outcomes, for the best models of prediction, is shown in Figure 3.

The participants' baseline demographic and clinical characteristics are shown in Table 1, considering subgroups of newborns with and without RDS in the birth scenarios of the study. Regarding prenatal data, newborns with RDS had a higher frequency of mothers with diabetes (*p* < 0.001) and hypertensive disease (*p* < 0.001) in birth scenario 1, but not in scenario 2 (*p* = 0.086 and *p* = 0.453, respectively). An important baseline characteristic to highlight is the no-RDS subgroup profile with high maternal disease frequency, ventilatory support, and NICU admission. For instance, the no-RDS subgroup of LBW newborns in the validation scenario comprised 102 (53.1%) newborns with mothers affected by hypertensive diseases and 115 (59.6%) newborns admitted to NICU. In both scenarios, children with RDS had higher ACTMF exposition (*p* < 0.001), lower gestational age (*p* < 0.001), lower birth weight (*p* < 0.001), and lower first-minute Apgar score (*p* < 0.001) than those without RDS.

**Table 1:** Baseline demographic and clinical characteristics of the pregnancy and newborns of the baseline and validation cohorts.

	Birth scenario 1 (modeling) Brazil (*n* = 780, 100%)				Birth scenario 2, LBW^b^ (validation) Brazil (*n* = 128, 42.0%); Mozambique (*n* = 177, 58.0%)				Comparison between scenarios
Characteristics	Total (*n* = 780)	RDS (*n* = 215)	No-RDS (*n* = 565)	*p*-value	Total (*n* = 305)	RDS (*n* = 112)	No-RDS (*n* = 193)	*p*-value	*p*-value
Prenatal conditions									
ACTFM, *n*/*N* (%)	273 (35.1)	184 (86.0)	89 (15.8)	<0.001^#^	141 (46,4)	86 (77.5)	55 (28.5)	<0.001^#^	<0.001^#^
Mother with diabetes, *n*/*N* (%)	125 (16.0)	54 (25.1)	71 (12.6)	<0.001^#^	20 (6.6)	11 (9.8)	9 (4.7)	0.086^#^	<0.001^#^
Mother with hypertensive disease, *n*/*N* (%)	169 (21.7)	80 (37.2)	89 (15.8)	<0.001^#^	156 (51.5)	54 (48.6)	102 (53.1)	0.453^#^	<0.001^#^
Rupture of membranes more than 18 h, *n*/*N* (%)	91 (11.7)	39 (18.1)	52 (9.3)	0.001^#^	41 (13.5)	22 (19.8)	19 (9.9)	0.015^#^	0.421^#^
Childbirth									
Reference gestational age at birth (weeks), median (IQR)	37.3 (6.3)	31.1 (4.4)	39.0 (3.4)	<0.001**	34.3 (3.5)	31.7 (3.5)	35.9 (3.3)	<0.001*	<0.001*
Preterm^a^, *n*/*N* (%)	366 (46.9)	214 (99.5)	152 (26.9)	<0.001^#^	234 (76.7)	109 (97.3)	125 (64.8)	<0.001^#^	<0.001^#^
Birth weight (g), median (IQR)	2,740 (1496)	1,360 (870)	3,085 (823)	<0.001**	1,930 (687)	1,385 (771)	2,075 (430)	<0.001*	<0.001*
Low-birth-weight^b^, *n*/*N* (%)	325 (41.7)	211 (98.1)	114 (20.2)	<0.001^#^	305 (100)	112 (100)	193 (100)	–	–
Very-low-birth-weight^c^, *n*/*N* (%)	136 (17.4)	125 (58.1)	11 (1.9)	<0.001^#^	73 (23.9)	65 (58.0)	8 (4.1)	<0.001^#^	0.015^#^
Sex, male, *n*/*N* (%)	389 (50.1)	113 (52.6)	276 (48.8)	0.355^#^	131 (43.0)	54 (51.8)	116 (60.1)	0,157^#^	0.033^#^
Anthropometric reference^d^				<0.001^#^				0.001^#^	<0.001^#^
• Small for gestational age, *n*/*N* (%)	114 (14.6)	55 (25.6)	59 (10.4)		139 (45.6)	73 (65.2)	82 (42.5)		
• Appropriate for gestational age, *n*/*N* (%)	607 (77.8)	154 (71.6)	453 (80.2)		155 (50.8)	35 (31.3)	104 (53.9)		
• Large for gestational age, *n*/*N* (%)	59 (7.6)	6 (2.8)	53 (9.4)		11 (3.6)	4 (3.6)	7 (3.6)		
1-min Apgar score, median (IQR)	8 (1)	7 (3)*	9 (1)	<0.001**	7 (2)	7 (2)	7 (1)	0.037**	<0.001*
5-min Apgar score, median (IQR)	9 (1)	9 (1)	9 (1)	<0.983**	9 (1)	9 (2)	9 (1)	0.653**	<0.001*
Resuscitation steps: initial, *n*/*N* (%)	384 (49.4)	202 (94.0)	182 (32.4)	<0.001^#^	152 (50.8)	87 (77.7)	65 (34.8)	<0.001^#^	<0.001^#^
Resuscitation steps: PPV, *n*/*N* (%)	155 (19.9)	105 (48.8)	50 (8.8)	<0.001^#^	59 (19.5)	44 (39.6)	15 (7.9)	<0.001^#^	0.844^#^
Resuscitation steps: Intubation at birth, *n*/*N* (%)	49 (6.3)	42 (19.5)	7 (1.2)	<0.001^#^	12 (4.0)	11 (9.8)	1 (0.5)	<0.001^#^	0.131^#^
Resuscitation steps: drugs, *n*/*N* (%)	2 (0.3)	1	0	–	3 (1.0)	3 (2.7)	0	–	–
72 h of life follow-up									
NICU admission, *n*/*N* (%)	239 (30.6)	210 (97.7)	70 (12.4)	<0.001^#^	225 (73.8)	110 (98.2)	115 (59.6)	<0.001^#^	<0.001^#^
Surfactant, *n*/*N* (%)	112 (14.4)	112 (52.1)	0	<0.001^#^	41 (13.4)	41 (36.6)	0	<0.001^#^	0.697^#^
Ventilatory support: CPAP, *n*/*N* (%)	250 (32.1)	181 (84.2)	69 (12.2)	<0.001^#^	128 (42.0)	97 (86.6)	31 (16.1)	<0.001^#^	0.002^#^
Ventilatory support: other noninvasive ventilation, *n*/*N* (%)^e^	56 (7.2)	55 (25.6)	1 (0.2)	<0.001^#^	37 (12.1)	32 (28.6)	5 (2.6)	<0.001^#^	0.009^#^
Ventilatory support: mechanical ventilation, *n*/*N* (%)	95 (12.2)	87 (40.5)	8 (1.4)	<0.001^#^	36 (11.8)	33 (29.7)	3 (1.6)	<0.001^#^	0.864^#^
Newborn mortality, *n*/*N* (%)	15 (1.0)	15 (7.0)	0	<0.001^##^	20 (6.6)	18 (16.1)	2 (1.0)	<0.001^##^	<0.001^##^

ACMF, antenatal corticosteroid therapy for fetal maturation; CPAP, continuous positive airway pressure; IQR, interquartile range; LBW, low birth weight; NICU, neonatal intensive care unit; NTT, transient tachypnea of the newborn; PPV, positive-pressure ventilation; RDS, respiratory distress syndrome.

^a^ Less than 37 weeks.

^b^ birth weight <2.5 kg.

^c^ birth weight <1.5 kg.

^d^ According to Intergrowth 21st.

^e^ Hood, nasal cannula, face mask and Biphasic Positive Airway Pressure.

* Mann Whitney *U* Test.

^#^ Chi-square.

^##^ Likelihood ratio chi-square statistic.

Comparing birth scenarios, the newborns had similar characteristics concerning rupture of membranes more than 18 h (*p* = 0.421), positive-pressure ventilation (*p* = 0.844), intubation at birth (*p* = 0.131) surfactant resuscitation steps, (*p* = 0.697), and mechanical ventilation (0.864) until 72 h of life. However, the LBW newborns in the birth scenario 2 had higher morbidity and mortality rates (*p* < 0.001) than newborns in the birth scenario 1.

Despite the primary outcome being RDS prediction, we still provided a more detailed analysis in the Supplementary Material, comparing three subgroups: RDS newborns, TTN newborns, and newborns without RDS or TTN.

### Primary outcome

**Figure 4:**
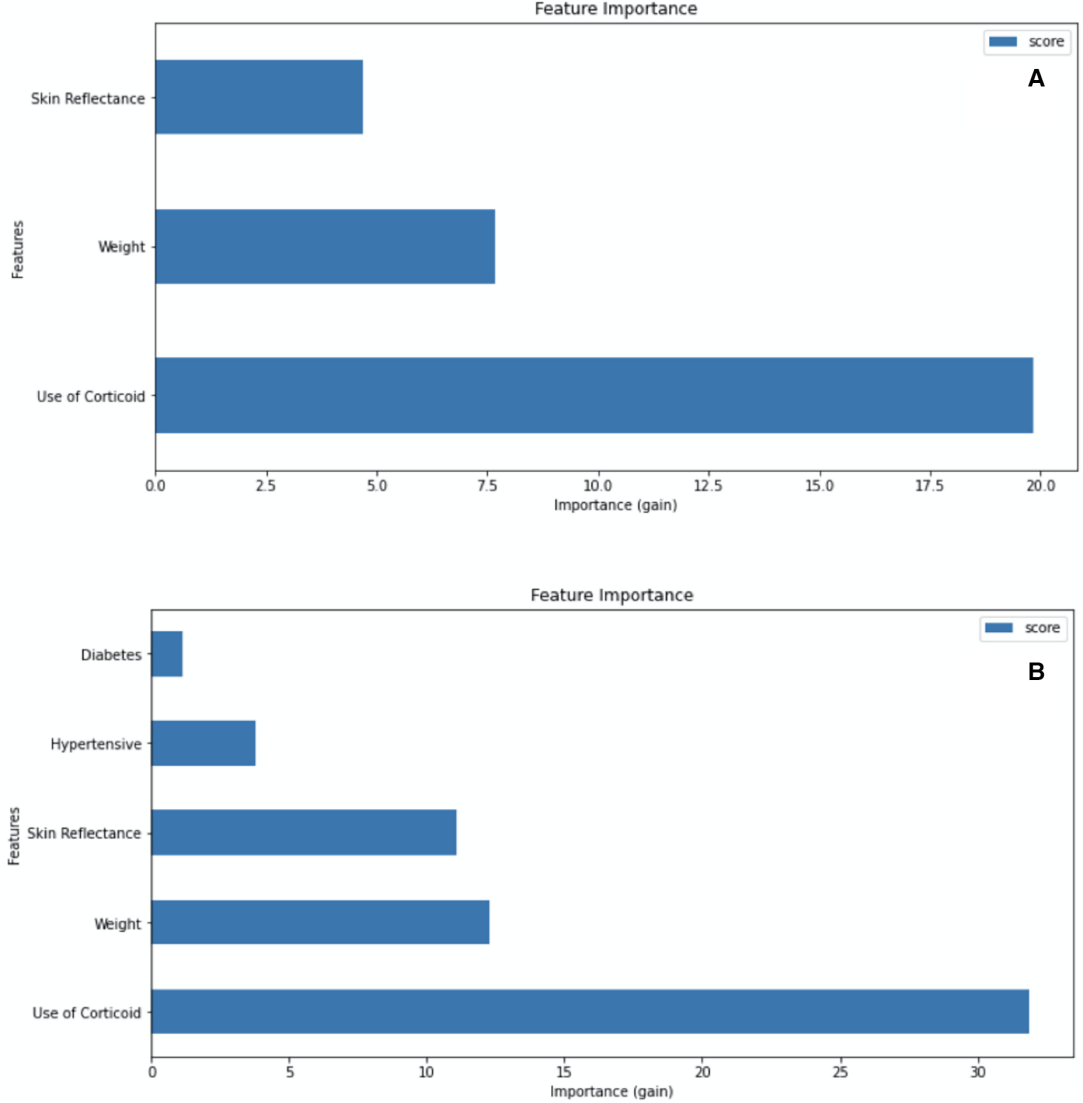
Attribute importance given by XGBoost when considering information gain that a variable brings when inserted into the model. (**A**) Model 1: trained with skin reflection + birth weight + Antenatal corticosteroid therapy for lung maturation, for the binary outcome RDS vs. non-RDS. (**B**) Model 2: trained with Skin reflection + birth weight + Antenatal corticosteroid therapy for lung maturation + diabetes + hypertensive diseases for the binary outcome RDS vs. non-RDS.

The machine learning modeling incorporated combinations of maternal and newborn characteristics associated with RDS to develop predictive algorithms that are useful at birth. Analyzing the importance feature given by XGBoost (Figure 4), and metrics of accuracy, precision, and recall (Supplementary Material), we consider the gain insufficient when maternal disease variables were inserted into the model. Models including hypertensive disease and diabetes data for the binary outcome for RDS had similar accuracy and F1 scores to models with the three baseline variables: skin reflection, birth weight, and ACTMF. The ACTMF was the variable with the highest importance in predicting RDS, followed by birth weight and skin reflection acquired by the optical component of the medical device in model 1 and model 2 (Figure 4).

In relation to discriminating among RDS, TTN, and neither of them using three classes of outcome modeling (models 3 and 4, Supplementary Material), the performance was worse than binary RDS yes/no prediction (models 1 and 2, Supplementary Material). When applying the models in the scenario of LBW newborns for external validation, metrics of prediction performance confirmed the advantages of the three-variable model with a binary RDS yes or no outcome, with an accuracy of 89.4% (95% CI: 88.6 to 90.3) and 82.3% in the cross-validation and external validation, respectively (model 1, Supplementary Material). As detailed in Supplementary Material, we chose the most parsimonious models for complete accuracy analysis.

There were no adverse events when performing the index test. The prediction accuracy of the test using the medical device at birth for RDS occurrence until 72 h of life is detailed in Table 2. Using cross-validation in the birth scenario used for modeling, algorithms with three or five independent variables delivered similar predictions regarding RDS discrimination, 89.7% (95% CI: 87.4 to 91.8) and 89.4% (95% CI: 87.0 to 91.4), respectively. Such accuracy occurred with high sensitivity and specificity, and the likelihood ratio for RDS was increased by approximately 10 times when the index test was positive. According to LBW and very-LBW newborns subgroup analysis, RDS prediction occurred with a high accuracy of 91.9% (95% CI: 86.0 to 95.9) despite a low specificity of 9.1% (95% CI: 0.23 to 41.3) when using model 1. Model 2, obtained with five variables, had no utility for RDS prediction in very-LBW newborns.

**Table 2:** Accuracy for respiratory distress syndrome during the first 72 h of life, according to the predictive algorithms with binary outcomes.

	Birth scenario 1—cross-validation (*n* = 780)		Birth scenario 2, LBW—external validation (*n* = 305)	
	Model 1 (skin reflection, BW, ACTMF)	Model 2 (skin reflection, BW, ACTMF, DB, HD)	Model 1 (skin reflection, BW, ACTMF)	Model 2 (skin reflection, BW, ACTMF, DB, HD)
Occurrence in overall group	RDS 215/780 (27.6%)	RDS 215/780 (27.6%)	RDS 112/305 (36.7%)	RDS 112/305 (36.7%)
All sample	Value (95% CI)	Value (95% CI)	Value (95% CI)	Value (95% CI)
ACU (%)	89.7 (87.4 to 91.8)	89.4 (87.0 to 91.4)	–	–
SEN (%)	85.6 (80.2 to 90.0)	84.7 (79.1 to 89.2)	–	–
SPE (%)	91.3 (88.7 to 93.5)	91.2 (88.5 to 93.4)	–	–
VPP (%)	79.0 (74.1 to 83.2)	78.5 (73.5 to 82.7)	–	–
VPN (%)	94.3 (92.3 to 95.8)	94.0 (91.9 to 95.5)	–	–
LR+	9.87 (7.51 to 12.97)	9.57 (7.30 to 12.54)	–	–
LR-	0.16 (0.11 to 0.22)	0.17 (0.12 to 0.23)	–	–
Occurrence in LBW	RDS 211/325 (64.9%)	RDS 211/325 (64.9%)	RDS 112/305 (36.7%)	RDS 112/305 (36.7%)
	Value (95% CI)	Value (95% CI)	Value (95% CI)	Value (95% CI)
ACU (%)	76.6 (71.6 to 81.1)	75.7 (70.7 to 80.3)	82.3 (77.5 to 86.4)	82.3 (77.5 to 86.4)
SEN (%)	87.2 (81.9 to 91.4)	86.3 (80.9 to 90.6)	82.1 (73.8 to 88.7)	79.5 (70.8 to 86.5)
SPE (%)	57.0 (47.4 to 66.3)	56.1 (46.5 to 65.4)	82.4 (76.3 to 87.5)	83.9 (78.0 to 88.8)
VPP (%)	79.0 (75.1 to 82.4)	78.5 (74.6 to 81.9)	73.0 (66.3 to 78.8)	74.2 (67.2 to 80.1)
VPN (%)	70.7 (62.1 to 78.0)	68.8 (60.3 to 76.3)	88.8 (84.2 to 92.2)	87.6 (83.0 to 91.1)
LR+	2.03 (1.63 to 2.52)	1.97 (1.59 to 2.44)	4.66 (3.40 to 6.40)	4.95 (3.54 to 6.92)
LR-	0.22 (0.15 to 0.33)	0.24 (0.17 to 0.36)	0.22 (0.14 to 0.32)	0.24 (0.17 to 0.35)
Occurrence in VLBW	RDS 125/136 (91.9%)	RDS 125/136 (91.9%)	RDS: 65/73 (89.0%)	RDS: 65/73 (89.0%)
	Value (95% CI)	Value (95% CI)	Value (95% CI)	Value (95% CI)
ACU (%)	91.9 (86.0 to 95.9)	91.2 (85.1 to 95.4)	84.9 (74.6 to 92.2)	84.9 (74.6 to 92.2)
SEN (%)	99.2 (95.6 to 100)	99.2 (95.6 to 100)	93.9 (85.0 to 98.3)	93.9 (85.0 to 98.3)
SPE (%)	9.1 (0.23 to 41.3)	0 (0.0 to 28.5)	12.5 (0.32 to 52.7)	12.5 (0.32 to 52.7)
VPP (%)	92.5 (91.1 to 93.7)	91.9 (91.7 to 92.0)	89.7 (86.9 to 91.9)	89.7 (86.9 to 91.9)
VPN (%)	50 (6.3 to 93.7)	0	22.0 (3.1 to 66.3)	22.0 (3.1 to 66.3)
LR+	1.09 (0.90 to 1.32)	0.99 (0.98 to 1.01)	1.07 (0.82 to 1.40)	1.07 (0.82 to 1.40)
LR−	0.09 (0.01 to 1.31)	–	0.49 (0.06 to 3.88)	0.49 (0.06 to 3.88)

ACU, accuracy; ACTFM, antenatal corticosteroid therapy for fetal maturation; BW, birth weight; DB, diabetes; CI, confidence interval; HD, hypertensive disease; LBW, low-birth-weight; LR+, likelihood ratio positive; likelihood ratio negative; LR-. SEN, sensibility; SPE, Specificity; NPV, negative predictive value; PPV, positive predictive value; VLBW, very-low-birth-weight.

Using the models for external validation in LBW newborns, algorithms with or without maternal diseases included had similar performance in predicting RDS as RDS occurrence was correctly predicted in 82% of newborns (95% CI: 77.5 to 86.4). The likelihood ratio for RDS increased approximately five times when the index test was positive (Table 2). Regarding the subgroup analysis of very-LBW newborns, global accuracy was similar to the overall group: 84.9% (95% CI: 74.6 to 92.2) for the model with or without maternal diseases as predictors.

**Figure 5:**
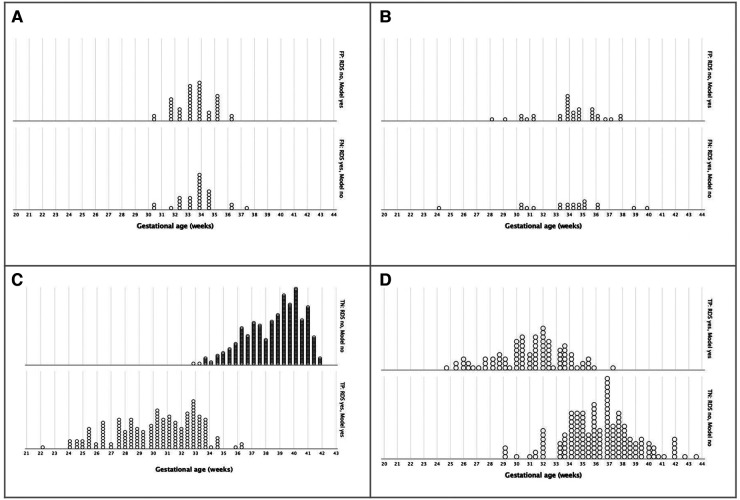
Confusion matrix for Respiratory Distress Syndrome prediction until 72 hours of life, according to gestational age at birth, using a three-variable-mode. (**A**) Incorrect prediction in birth scenario 1 - Cross-validation (*n* = 780). (**B**) Incorrect prediction in birth scenario 2, LBW - External validation (*n* = 305). (**C**) Correct prediction in birth scenario 1 - Cross-validation (*n* = 780). (**D**) Correct prediction in birth scenario 2, LBW - External validation (*n* = 305).

Analyzing the confusion matrix for RDS prediction according to gestational age at birth (Figure 5), we found false positives and false negatives more frequently around 33 and 34 weeks of gestation in both birth scenarios. However, it is relevant to notice that, in external validation, the three-variable model (model 1) discriminated most of the LBW newborns with (true positive) and without (true negative) RDS in the range of 29 to 37 weeks of gestation.

In order to inspect similarities and differences between newborns with or without correct RDS prediction, we compared the clinical characteristics in the validation scenario (Supplementary Material). Gestational age, birth weight, maternal diseases, and TTN occurrence were statistically similar between subgroups. Only NICU admission within the first 72 h occurred more frequently in newborns with an incorrect prediction (90.7% vs. 70.9%, *p* = 0.002).

## Discussion

### Main findings

Improving healthcare equity is a primary goal of the United Nations — this aim makes the reduction of infant mortality a priority (27). Digital health, including affordable and valuable medical devices and artificial intelligence, has brought hope to improve health for everyone (28, 29). The main outcome of the present study was providing a promissory predictive model using a medical device with an AI algorithm inside. Of every 100 newborns assessed, 90 were correctly classified as a higher risk or not for RDS until 72 h of life, considering the dataset that provides predictive models. The prediction accuracy remained high in the LBW newborns that composed the validation scenario, 82 in every 100, where the RDS and other neonatal morbidities and mortality were more frequent than in the model development scenario.

The same sort of study has been presented, integrating computational technology to identify predictors of neonatal mortality, such as the lecithin and sphingomyelin ratio by machine learning applied to mild-infrared spectra (30) or acoustic features of the crying of newborns (31). Reviews have highlighted the importance of birth weight, Apgar score, and antenatal steroids (28). Our approach has the advantage of using only three predictive variables obtained from a prospective temporality clinical trial approach to provide prediction before the disease occurrence. Models with five predictive variables, including maternal diseases (i.e., diabetes and hypertensive diseases) did not show advantages over models based on skin maturity optical assessment, birth weight, and steroids. This finding will certainly facilitate the use of the device by caregivers who deliver care at birth in LMICs.

### Comparisons and subgroups of analysis

Considering the very-LBW subgroup of analysis, our results with a three-variables predictive model achieved an accuracy of 84.9% (95% CI, 74.6 to 92.2). In comparison, using an extensive historical 14-year inpatient dataset and many predictive variables, Jaskari et al. classified bronchopulmonary dysplasia in a retrospective dataset of very-LBW, with an accuracy of around 0.899 AUROC (32). Furthermore, analyzing a prospective dataset of newborns older than 24 weeks of gestation, our modeling achieves an accuracy of 89.7% (95% CI, 87.4 to 91.8), while Betts et al. reported RDS prediction with an accuracy of 0.923 (0.917, 0.928) among inpatients younger than 39 weeks of gestation (33), using the same dataset as Jaskari et al. (32). So far, our study is the first that has used a physical measurement of skin maturity, previously described (16, 19, 20), using a prospective dataset from clinical trials with nearly similar accuracy to other more complex models.

Early detection of severe neonatal morbidities such as RDS is critical to halt disease progression and prevent further complications or death. Risk identification of the occurrence might provide means for opportune diagnosis and due care with surfactant access, enhancing chances of survival with minimal sequelae, even with the referral of newborns (5). In LMICs, the availability of a NICU in a center of excellence is often far from the place of birth of this preterm infant (4). The limited number of intensive care beds that can receive real RDS-risk newborns justifies a reliable and helpful predictive test to support low-risk newborns' retention decisions, optimizing resources. By analyzing the confusion matrix, the outcome of the present study showed early and promising discrimination of RDS even in late preterm newborns in the development and LBW validation scenarios.

Worldwide, hard decisions in scenarios with scarce resources are taken daily based on birth weight, with particular attention to late preterm births that account for most preterm births (34). Birth weight is the most accessible and significant determinant of the likelihood of survival at birth, but it alone is not enough to predict neonatal outcomes. Placental dysfunction, maternal-fetal conditions affecting lung maturation such as smoking, cardiovascular diseases, and prenatal exposure to drugs such as steroids are also determinants (35). Known antenatal predictors of RDS, such as prenatal Doppler velocimetry and the lamellar body count test on gastric aspirates have limitations in LMICs due to high costs and a lack of professionals with the necessary skills (8, 36).

### Implications for practice and the role of the index test

The role of the index test used to predict RDS might be a prompt risk indication immediately at birth, anticipating best practices of management in scenarios with limited resources or optimizing access to existing facilities. This study is a premarket approach using data from two clinical trials to validate the algorithm for real-time RDS prediction at birth. The skin reflection can be acquired from the device, and the user quickly introduces some clinical variables, as presented in Figure 1. Facilities without neonatologists, mobile emergency services, and caregivers in primary units where a preterm birth can occur are the potential targets of this device. The approach is intended to quickly offer a prediction based on variables easily accessible at birth scenarios added to the skin maturity assessment, even outside hospitals. In the same way, a professional in maternity and NICU settings could be interested in this prediction to manage clinical follow-up of newborns and bed occupancy.

Despite recent advances in the perinatal management of RDS, controversies still exist. Lower emphasis on radiographic diagnosis and classification of RDS, such as ground glass with air bronchograms, directs management toward a preventive surfactant treatment approach. Definitions based on blood gas analyses are also redundant, as management has moved towards a preventive surfactant treatment approach based on clinical assessment of the work of breathing and oxygen requirement to avoid worsening the syndrome. Current RDS management aims to maximize survival by minimizing complications such as air leaks and bronchopulmonary dysplasia (5).

### Sources of potential bias and generalizability

Despite the development of a new technology that allows skin maturity associated with birth data to be used as a marker of lung maturity, sources of potential bias can limit the generalizability of the outcomes. The development and validation scenarios had relevant differences regarding RDS frequency in newborns, morbidity, and mortality. Moreover, the accuracy of the machine learning models was sustained by a high specificity of 91.3% (95% CI, 88.7 to 93.5). In false-positive RDS prediction in LBW newborns, unnecessary interventions such as transferring to a referral center can occur in approximately 18% of newborns. Nonetheless, assuming the implementation of a screening test, a point-of-care prediction in conjunction with clinical protocols, this approach has the potential to enhance neonatal care. Future studies are necessary to measure the influence of disease incidence on generalizing the models, as in the primary care birth scenario or low complexity hospitals where the incidence of preterm birth and RDS is lower than ours. The performance of the prediction in the subgroups analysis considering ranges of gestational age and birth weight might still require further large samples.

Regarding skin maturity importance in the model, the rationale which relies on a direct relationship between epidermal barrier competence and neonatal survival faces limitations after 35 weeks of gestation, when the epidermis is complete (37). Therefore, the test may perform better in preterm newborns than in term newborns; similar to previous studies, we used the device to predict gestational age (38). Finally, there is a potential bias associated with suboptimal pregnancy dating in the validation scenario since the inclusion criteria admitted obstetric ultrasound examinations before 24 weeks or just using a reliable last menstrual period, which has already been reported (38). At the same time, data from the clinical trials in Brazil and Mozambique provided a picture of using the test under natural conditions with barriers to high-cost technologies.

## Conclusions

The objective measurement of skin maturity alongside machine learning models opens new opportunities to recognize complex patterns among variables in RDS outcome prediction. The models adjusted for skin reflection, birth weight, and ACTMF at birth as RDS predictors for 72 h of life achieved high accuracy in developing and validating modeling using clinical trial datasets. This study demonstrates a new way to assess neonatal lung immaturity, providing potential opportunities for more effective and early caring with an automated medical device tester.

## Data Availability

The data analyzed in this study is subject to the following licenses/restrictions: Data is available upon reasonable request and after anonymization to ensure ethical and legal data sharing, thus preserving the confidentiality of the persons who participated in this study. Requests to access these datasets should be directed to zilma@ufmg.br.
