# Supplementary material for: Respiratory distress syndrome prediction at birth by optical skin maturity assessment and machine learning models for limited-resource settings: a development and validation study
Source: Front Pediatr. 2023 Nov 15;11:1264527. doi: 10.3389/fped.2023.1264527 (PMC10694507; doi:10.3389/fped.2023.1264527)
Supplement: Supplementary file 1 [file Table1.docx]

Supplementary Table 1. Machine learning modeling for respiratory distress syndrome prediction at birth with follow-up until 72h of life

| **Model** | **Outcome** | **Birth scenario 1**  (cross-validation, n=780) | | **Birth scenario 2**  (external validation, n=305) | |
| --- | --- | --- | --- | --- | --- |
|  |  | ACU (%)  (95% CI) | F1 score  (95% CI) | ACU (%) | F1 score |
| **Model 1**: Skin reflection + BW + ACTMF | RDS vs. non-RDS | 89.4  (88.6 to 90.3) | 89.6  ( 88.7 to 90.4) | 82.3 | 82.5 |
| **Model 2**: Skin reflection + BW + ACTMF+ DB + HD | RDS vs. non-RDS | 89.6  (88.7 to 90.5) | 89.7  ( 88.8 to 90.6) | 82.3 | 82.4 |
| **Model 3:** Skin reflection + BW + ACTMF | RDS vs. TTN vs. None | 85.0  (84.2 to 85.7) | 81.8  (81.1 to 82.5) | 64.2 | 57.6 |
| **Model 4**: Skin reflection + BW + ACTMF+ DB + HD | RDS vs. TTN vs. None | 84.8  (84.2 to, 85.4) | 81.5  ( 80.9 to 82.1) | 60.6 | 53.2 |

ACU: accuracy for respiratory distress syndrome. ACTMF: Antenatal corticosteroid therapy for lung maturation. BW: birth weight. DB: diabetes. CI: confidence interval. HD: hypertensive disease. TTN: transient tachypnea of the newborn. RDS: Respiratory distress syndrome.

Supplementary Table 2. Baseline demographic and clinical characteristics of newborns of the baseline and validation cohorts, according to respiratory distress syndrome and newborn transient tachypnea diagnosis

|  | **Birth scenario 1**  Brazil (n=780, 100%) | | | | | **Birth scenario 2, LBW**  Brazil (n=128, 42.0%); Mozambique (n=177, 58.0%) | | | | |
| --- | --- | --- | --- | --- | --- | --- | --- | --- | --- | --- |
| **Characteristics** | **Total**  (n=780) | **RDS** (n=215) | **TTN** (n=70) | **None** (n=495) | **p-value** | **Total**  **(n=305)** | **RDS** (n=112) | **TTN** (n=67) | **None** (n=126) | **p-value** |
| Reference gestational age at birth (weeks), median (IQR) | 37.3 (6.3) | 31.1 (4.4) | 35.2 (2.6) | 39.2 (2.9) | <0.001** | 34.3 (3.5) | 31.7 (3.5) | 34.6 (2.5) | 36.5 (2.7) | <0.001* |
| Preterm^a^, n/N (%) | 366 (46.9) | 214 (99.5) | 58 (82.9) | 94 (19.0) | <0.001^#^ | 234 (76.7) | 109 (97.3) | 54 (80.60 | 71 (56.3) | <0.001^#^ |
| Birth weight (g), median (IQR) | 2740 (1496) | 1360* (870) | 2202* (883) | 3179* (710) | <0.001** | 1930 (687) | 1385 (771) | 1940 (380) | 2160 (420) | <0.001* |
| Low-birth-weight^b^, n/N (%) | 325 (41.7) | 211 (98.1) | 51 (72.9) | 63 (12.7) | <0.001^#^ | 305 (100) | 112 (100) | 67 (100) | 126 (100) | … |
| Very-low-birth-weight^c^, n/N (%) | 136 (17.4) | 125 (58.1) | 6 (8.6) | 5 (1) | <0.001^#^ | 73 (23.9) | 65 (58.0) | 3 (4.5) | 4 (4.0) | <0.001^#^ |
| Sex, male, n/N (%) | 389 (50.1) | 113 (52.6) | 37 (52.9) | 239 (48.3) | 0.504^#^ | 131 (43.0) | 54 (48.2) | 30 (44.8) | 47 (37.3) | 0.223^#^ |
| Anthropometric reference^d^ |  |  |  |  | <0.001^#^ |  |  |  |  | <0.001^#^ |
| Small for gestational age, n/N (%) | 114 (14.6) | 55 (25.6) | 12 (17.1) | 47 (9.5) |  | 139 (45.6) | 73 (65.2) | 36 (53.7) | 46 (36.5) |  |
| Appropriate for gestational age, n/N (%) | 607 (77.8) | 154 (71.6) | 55 (78.6) | 398 (80.4) |  | 155 (50.8) | 35 (31.3) | 27 (40.3) | 77 (61.1) |  |
| Large for gestational age, n/N (%) | 59 (7.6) | 6 (2.8) | 3 (4.3) | 50 (10.1) |  | 11 (3.6) | 4 (3.6) | 4 (6.0) | 3 (2.4) |  |
| ACTFM, n (%) | 273 (35.1) | 184 (86.0) | 37 (52.9) | 52 (10.5) | <0.001^#^ | 141 (46,4) | 86 (77.5) | 25 (37.3) | 30 (23.8) | <0.001^#^ |
| Mother with diabetes, n/N (%) | 125 (16.0) | 54 (25.1) | 16 (22.9) | 55 (11.1) | <0.001^#^ | 20 (6.6) | 11 (9.8) | 4 (6.0) | 5 (4.1) | 0.202^#^ |
| Mother with hypertensive disease, n/N (%) | 169 (21.7) | 80 (37.2) | 25 (35.7) | 64 (12.9) | <0.001^#^ | 156 (51.5) | 54 (48.6) | 38 (56.7) | 64 (51.6) | 0.578^#^ |
| Rupture of membranes more than 18 hours, n/N (%) | 91 (11.7) | 39 (18.1) | 18 (25.7) | 34 (6.9) | <0.001^#^ | 41 (13.5) | 22 (19.8) | 10 (14.9) | 9 (7.2) | 0.017^#^ |
| 1-minute Apgar score, median (IQR) | 8 (1) | 7 (3)* | 8 (1) | 9 (1) | <0.001** | 7 (2) | 7 (2) | 7 (2) | 8 (1) | 0.010** |
| 5-minute Apgar score, median (IQR) | 9 (1) | 9 (1) | 9 (1) | 9 (1) | <0.983** | 9 (1) | 9 (2) | 8 (2) | 9 (1) | 0.047** |
| Resuscitation steps: initial, n/N (%) | 384 (49.4) | 202 (94.0) | 55 (78.6) | 127b(25.8) | <0.001^#^ | 152 (50.8) | 87 (77.7) | 27 (40.3) | 38 (31.7) | <0.001^#^ |
| Resuscitation steps: VPP, n/N (%) | 155 (19.9) | 105 (48.8) | 17 (24.3) | 33 (6.7) | <0.001^#^ | 59 (19.5) | 44 (39.6) | 4 (6.0) | 11 (8.9) | <0.001^#^ |
| Resuscitation steps: Intubation at birth, n/N (%) | 49 (6.3) | 42 (19.5) | 1 (1.4) | 6 (1.2) | <0.001^#^ | 12 (4.0) | 11 (9.8) | 1 (1.5) | 0 (0) | <0.001^#^ |
| Resuscitation steps: drugs, n/N (%) | 2 (0.3) | 1 | 0 | 1 | … | 3 (1.0) | 0 | 0 | 0 | … |
| NICU admission within first 72 hours, n/N (%) | 239 (30.6) | 210 (97.7) | 40 (57.1) | 3.0 (6.1) | <0.001^#^ | 225 (73.8) | 110 (98.2) | 61 (91.0) | 54 (42.9) | <0.001^#^ |
| Ventilatory support: CPAP, n/N (%) | 250 (32.1) | 181 (84.2) | 47 (67.1) | 22 (4.4) | <0.001^#^ | 128 (42.0) | 97 (86.6) | 24 (35.8) | 7 (5.6) | <0.001^#^ |
| Ventilatory support: other noninvasive ventilation, n/N (%) | 56 (7.2) | 55 (25.6) | 1 (1.4) | 0 | <0.001^#^ | 37 (12.1) | 32 (28.6) | 1 (1.5) | 4 (3.2) | <0.001^#^ |
| Ventilatory support: mechanical ventilation, n/N (%) | 95 (12.2) | 87 (40.5) | 8 (1.6) | 95 (12.2) | <0.001^#^ | 36 (11.8) | 33 (29.7) | 1 (1.5) | 2 (1.6) | <0.001^#^ |
| Newborn mortality within first 72 hours, n/N (%) | 15 (1.0) | 15 (7.0) | 0 | 0 | <0.001^##^ | 20 (6.6) | 18 (16.1) | 0 (0) | 2 (1.6) | <0.001^##^ |

ACMF: Antenatal Corticosteroid Therapy for Fetal Maturation. CPAP: Continuous Positive Airway Pressure. IQR: interquartile range. LBW: low birth weight, below 2.5Kg. NICU: neonatal intensive care unit. NICU: neonatal intensive care unit. TTN: transient tachypnea of the newborn. PPV: Positive-Pressure Ventilation. RDS: respiratory distress syndrome.^**^Kruskal Walis. ^#^Qui-square. ^##^Likelihood ratio chi-square statistic.

^a^Less than 37 weeks. ^b^birth weight <2.5kg.  ^c^birth weight <1.5kg. ^d^According to Intergrowth 21st.

Supplementary Table 3. Baseline demographic and clinical characteristics of newborns, according to respiratory distress syndrome prediction

|  | Correct SDR prediction  (n=251) | Incorrect SDR prediction  (n=54) | p-value |
| --- | --- | --- | --- |
| Reference gestational age at birth (weeks), median (IQR) | 34.4 (3.7) | 33.9 (2.7) | 0.281* |
| Birth weight (g), median (IQR) | 1930 (730) | 1878 (503) | 0.504* |
| Very-low-birth-weight^a^, n/N (%) | 62 (24.7) | 11 (20.4) | 0.458^#^ |
| Mother with hypertensive disease, n/N (%) | 131 (52.6) | 25 (46.3) | 0.400^#^ |
| Mother with diabetes, n/N (%) | 15 (6.0) | 5 (9.6) | 0.340^#^ |
| TTN diagnosis, n/N (%) | 51 (20.3) | 16 (29.6) | 0.134^#^ |
| NICU admission within first 72 hours, n/N (%) | 178 (70.9) | 49 (90.7) | 0.002^#^ |
| Newborn mortality within first 72 hours, n/N (%) | 18 (7.2) | 2 (3.7) | 0.644^#^ |

NICU: neonatal intensive care unit. NICU: neonatal intensive care unit.^*^Mann Whitney U Test. ^#^Chi-square Test. ^a^birth weight <1.5kg.
